# Supplementary material for: The Functional Identification of the CYP2E1 Gene in the Kidney of Lepus yarkandensis
Source: Int J Mol Sci. 2025 Jan 8;26(2):453. doi: 10.3390/ijms26020453 (PMC11764603; doi:10.3390/ijms26020453)
Supplement: Supplementary file 1 [file ijms-26-00453-s001.zip › ijms-3329799-supplementary.pdf]

Supplementary Figures

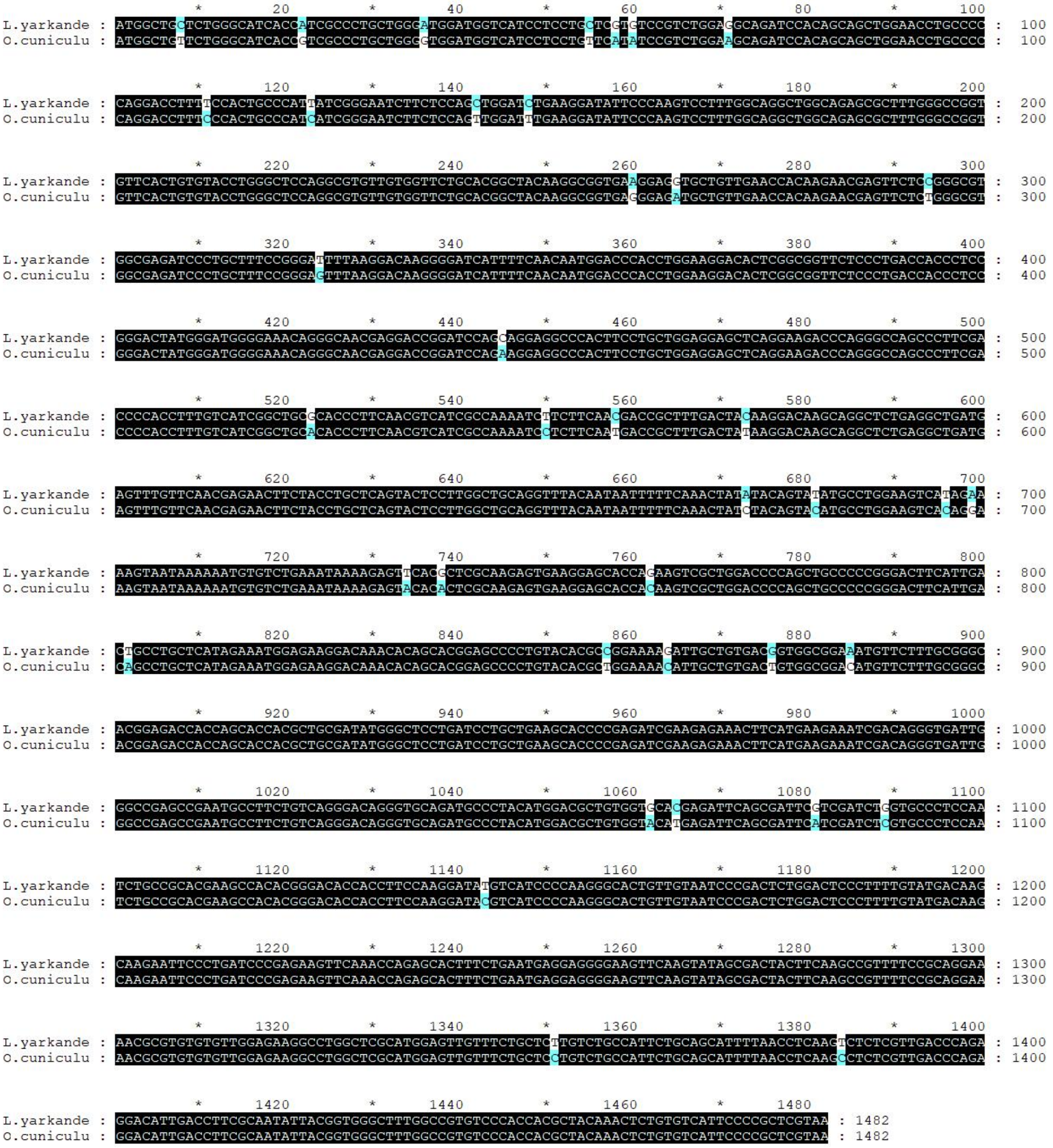

Figure S1. Comparison of the *CYP2E1* Gene Nucleotide Sequences between *L. yarkandensis* and *O.cuniculus*

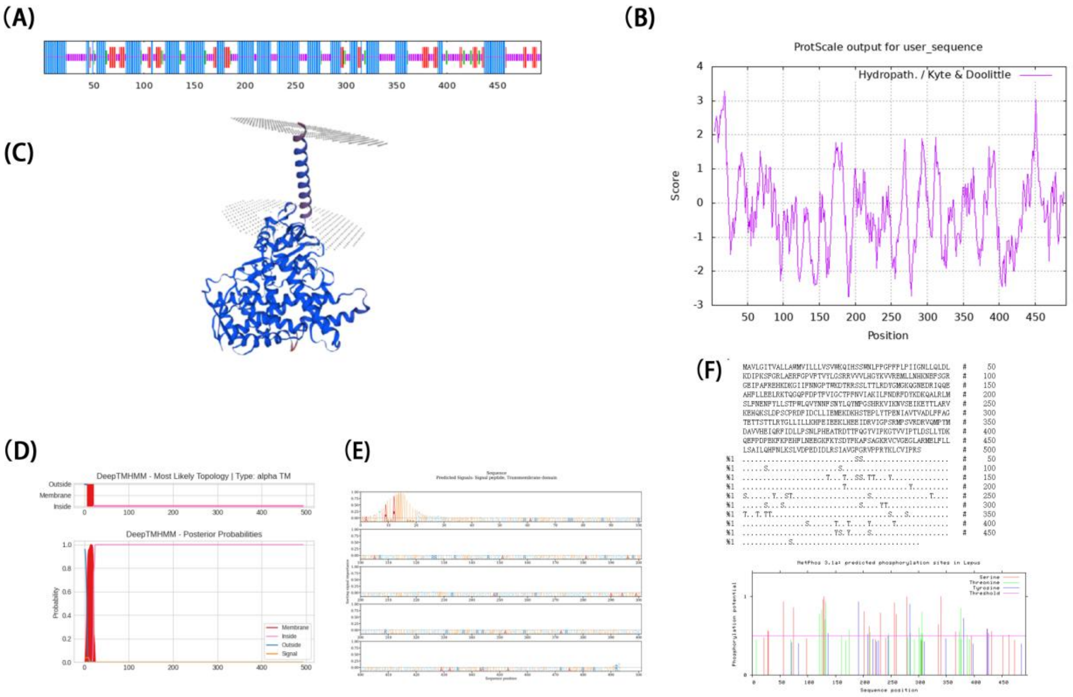

Figure S2. Functional Analysis of the CYP2E1 Protein from *L. yarkandensis*.(A) A secondary structure prediction of the CYP2E1 protein from its complete amino acid sequence. Long blue lines, short red lines, short green lines and short pink lines show predicted alpha helices, extended strands, beta turns and random coils, respectively. (B) Hydropathy characteristics of CYP2E1 protein. The hydropathy values of each amino acid are plotted, with positive and negative values indicating hydrophobicity and hydrophilicity, respectively. (C Prediction of the tertiary structure of CYP2E1 protein. The color shows the order of the peptide chain. (D) Prediction of transmembrane regions. (E) Prediction of subcellular localization. (F) Prediction of phosphorylation sites.

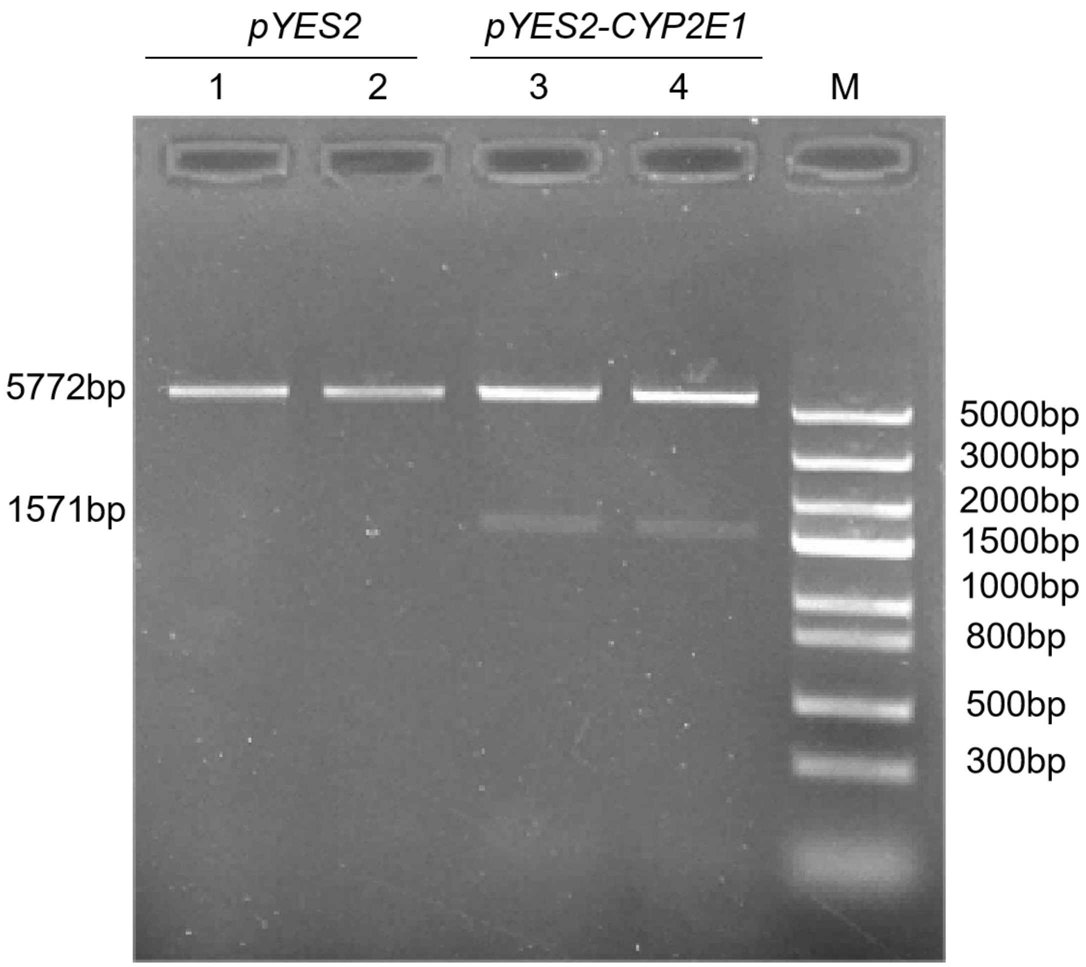

Figure S3. Double digestion identification of pYES2 and pYES2-*CYP2E1*.

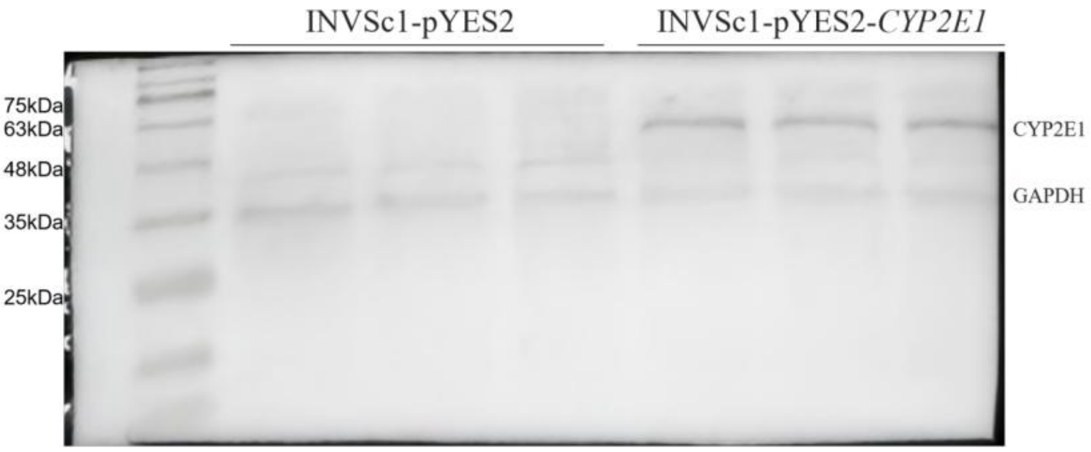

Figure S4. Western blotting detection of CYP2E1 protein expression levels in INVSc1-pYES2 and INVSc1-pYES2-*CYP2E1*.
